# Aldosterone and cortisol synthesis regulation by angiotensin-(1-7) and angiotensin-converting enzyme 2 in the human adrenal cortex

**DOI:** 10.1097/HJH.0000000000002816

**Published:** 2021-03-01

**Authors:** Brasilina Caroccia, Paul-Emmanuel Vanderriele, Teresa Maria Seccia, Maria Piazza, Livia Lenzini, Selene Prisco, Francesca Torresan, Oliver Domenig, Maurizio Iacobone, Marko Poglitsch, Gian Paolo Rossi

**Affiliations:** a Specialized Center for Blood Pressure Disorders-Regione Veneto and Emergency-Hypertension Unit, Department of Medicine-DIMED, University of Padua; b Endocrine Surgery Unit, Department of Surgery, Oncology and Gastroenterology, University of Padua, Padova, Italy; c Attoquant Diagnostics, Vienna, Austria

**Keywords:** angiotensin-(1–7), angiotensin-converting enzyme, angiotensin receptors, renin–angiotensin system, angiotensin-converting enzyme-2, aldosterone, primary aldosteronism

## Abstract

The branch of the renin--angiotensin system constituting angiotensin-(1–7) [Ang-(1–7)], the Ang II type 2 receptor, the Mas receptors and the Ang-(1–7)-forming enzyme ACE-2, by counteracting the Ang II type 1 receptor (AT1R)-mediated effects, are held to be cardiovascular protective in several conditions. However, whether Ang-(1–7) and ACE-2 are detectable in human adrenocortical tissues and whether they affect aldosterone and cortisol biosynthesis was unknown.

**Methods:**

We measured angiotensin peptides with liquid chromatography tandem-mass spectrometry and ACE-2 mRNA with digital droplet (dd)PCR in human aldosterone-producing adenoma (APA) and APA-adjacent tissue obtained from patients with primary aldosteronism. We also investigated the effects of Ang-(1–7) and the ACE-2 activator diminazene aceturate (DIZE) on aldosterone synthase (*CYP11B2*) and 11β-hydroxylase (*CYP11B1*) gene expression, in the absence or presence of the AT1R antagonist irbesartan, or of the MasR antagonist A779.

**Results:**

APA and APA-adjacent adrenocortical tissues express *ACE-2* mRNA and contain detectable amounts of Ang II and Ang-(2–8), but not of Ang I, Ang-(1–5), Ang (3–8) and Ang-(1–7). Under unstimulated and Ang II- stimulated conditions Ang-(1–7) did not blunt *CYP11B1* and *CYP11B2* mRNA. At supraphysiological concentrations (10^−4^ mol/l), Ang-(1–7) stimulated both *CYP11B1* and *CYP11B2* mRNA via the AT1R. The ACE-2 activator DIZE increased by 1.5-fold *ACE-2* mRNA but did not blunt Ang II- upregulated *CYP11B1* and *CYP11B2* expression.

**Conclusion:**

These results do not support the hypothesis that the ACE-2/Ang-(1–7)/MasR axis play a protective role by counteracting enhanced aldosterone secretion in humans.

## INTRODUCTION

The adrenocortical zona glomerulosa is the main site of expression of aldosterone synthase (CYP11B2), the key mitochondrial aldosterone-forming enzyme, and, therefore, entails the major source of this mineralocorticoid hormone [1,2]. More than a dozen factors regulate *CYP11B2* expression (reviewed in [3]), among which the octapeptide angiotensin (1–8), better known as Ang II, is regarded as one of the most important [4]. In primary aldosteronism, the most common endocrine form of arterial hypertension, excess aldosterone secretion was contended to be independent from renin and Ang II as the patients with primary aldosteronism show very low levels of Ang II in plasma [5,6]. However, the finding of renin in rat zona glomerulosa and in human aldosterone-producing adenoma (APA) [7,8], alongside the detection of angiotensin receptors in such tumors [9], suggested that locally generated angiotensin peptides can be functionally active in these tissues. Nonetheless, there was no information on the presence of Ang II and related angiotensin peptides in human adrenocortical tissues and in adrenal vein blood from primary aldosteronism patients so far.

The ‘alternative’ branch of the renin--angiotensin system constituting angiotensin-converting enzyme 2 (ACE-2) and its product angiotensin-(1–7) [Ang-(1–7)], which activates signaling pathways predominantly via the Mas (MasR) receptor, is held to play a protective role in cardiovascular diseases. This would occur by counteracting Ang II type 1 receptor (AT1R)-mediated vasoconstriction [10,11], cell proliferation and sodium-retaining actions [12], and by correcting endothelial dysfunction in experimental models of atherosclerosis, heart failure and inflammation [13]. Accordingly, a loss of these protective effects of Ang-(1–7) was found in rodents with cardiovascular diseases [14,15]. Thus far, human data were limited to the suggestion of a possible compensatory role of ACE-2, which originated from the higher serum levels found in hypertensive patients compared with healthy individuals [16].

Given that activation of ACE-2/Ang-(1–7)/MasR/AT2R pathway can protect from arterial hypertension and hypertension-mediated organ damage by counteracting Ang II/AT1R-mediated effects [17], we hypothesized that it could also be beneficial by blunting aldosterone production in human primary aldosteronism, the paradigm of aldosterone- and salt-dependent hypertension. This study was, therefore, set out to measure angiotensin peptides in plasma and adrenal tissues of primary aldosteronism patients and determine ACE-2 expression and Ang-(1–7) formation in APA and in APA-adjacent tissues obtained *ex vivo* at surgery. We also sought for ascertaining if Ang-(1–7) could blunt unstimulated or Ang II-enhanced CYP11B2 and if augmented endogenous Ang-(1–7) generation with diminazene aceturate (DIZE), allegedly an ACE-2-activator, affected aldosterone and cortisol biosynthesis under resting conditions and after Ang II-stimulation.

## MATERIAL AND METHODS

### Patients and tissues

We recruited patients with a conclusive diagnosis of primary aldosteronism because of an APA, as established by the ‘five corner criteria’ [3]. Their main clinical features at baseline and after adrenalectomy are shown in Supplemental Table 1. We obtained APA tissue and APA-adjacent tissue from informed consenting patients under sterile conditions in the operating room. After cutting the adrenal gland into halves following the equatorial plane of the APA, small pieces of the tumour and adjacent tissue were immediately snap-frozen in isopentane kept on dry ice and then stored in liquid nitrogen in the institutional human adrenal tissue tumour bank. The collection and the use of the adrenal tissues was approved by the Ethics Committee of the University Hospital of Padova (Protocol # 1925P).

### Angiotensin peptides in the adrenal gland and adrenal vein blood

Angiotensin peptides were measured in the adrenocortical tissues, in plasma collected during adrenal vein sampling (AVS) from each adrenal vein and from the infrarenal inferior vena cava. These measurements were blindly performed at Attoquant Diagnostics GmbH (Vienna, Austria) with liquid chromatography tandem-mass spectrometry analysis (LC-MS/MS), as described in detail [18]. The lower limits of detection for these assays are shown in Table 1.

**TABLE 1:** Angiotensin peptides in human adrenal gland and in adrenal vein blood from primary aldosteronism patients

	Adrenocortical tissues		Adrenal venous blood			
Peptides	APA-adjacent tissue (fmol/g)	APA tissue (fmol/g)	APA side (fmol/ml)	Contralateral side (fmol/ml)	APA side SI-corrected (fmol/ml)	Contralateral side SI-corrected (fmol/ml)
Angiotensin I (Ang 1–10)	ND	ND	16.5 (10.5–33.45)	7.1 (4.6–17.5)	0.5 (0.3–0.8)	0.3 (0.2–1.3)
Angiotensin II (Ang 1–8)	167.4^a^ (122.5–731.0)	71.2^b^ (25.0–132.7)	14.3 (7.4–23.3)	7.5 (3.3–22.2)	0.5 (0.3–0.9)	0.2 (0.1–0.9)
Ang (1–7)	ND	ND	ND	ND	–	–
Ang (2–8)	123.3^c^ (54.6–1078.4)	124.3^d^ –	ND	ND	–	–
Ang (1–5)	ND	ND	ND	ND	–	–
Ang (3–8)	ND	ND	ND	ND	–	–
Selectivity index	–	–	31.4 (6.4–46.6)	–	–	–
Lateralization index	–	–	19.0 (8.0–55.0)	–	–	–

The median levels of angiotensin peptides measured in adrenal vein blood were provided as raw data and also corrected for the dilution and the catheter's tip proximity to the adrenal cortex by normalizing for selectivity index (SI), calculated as the ratio of cortisol in the adrenal vein and in inferior vena cava blood. ND: not detectable. The lower limit of detection (LLOD) in tissues were: for angiotensin I (Ang 1–10) 40 fmol/g, for angiotensin II (Ang 1–8) 25 fmol/g, for Ang (1–7) 60 fmol/g, for Ang (2–8) 50 fmol/g, for Ang (1–5) 25 fmol/g, for Ang (3–8) 25 fmol/g. In blood, the corresponding values were: angiotensin I (Ang 1–10) 3 pmol/l, for angiotensin II (Ang 1–8) 1.5 pmol/l, for Ang (1–7) 3 pmol/l, for Ang (2–8) 2 pmol/l, for Ang (1–5) 1.5 pmol/l, for Ang (3–8) 1.5 pmol/l.

a This peptide was detectable in 7/10 samples.

b This peptide was detectable in 6/10 samples.

c This peptide was detectable in 3/10 samples.

d This peptide was detectable in 1/10 samples.

RAS equilibrium analysis was performed in human heparin plasma pool containing 50 ng/ml rhACE-2 (recombinant human ACE-2; R&D Systems, Vienna, Austria) in the presence and absence of 100 μg/ml DIZE (Sigma-Aldrich, Milan, Italy) in order to clarify if DIZE is an allosteric activator of ACE-2 as described [19].

### In-vitro cell experiments

Human adrenocortical cancer NCI-H295R cells (ATCC/LGC Standards S.r.L., Milan, Italy) were grown in RPMI medium supplemented with 10% fetal bovine serum (FBS), 1% glutamine and 1% antibiotic/antifungal mixture. They were seeded in 12-well plates at 2 × 10^5^ cells for well, grown to sub-confluence (80%) and starved for 24 h with RPMI medium supplemented with 0.5% FBS, 1% glutamine and 1% antibiotic/antimycotic before treatment. The cells were treated with DIZE at concentrations ranging from (10^–7^ to 10^–4^ mol/l), alone or in presence of (10^–7^ mol/l) Ang II (Sigma-Aldrich). NCI-H295R cells were also treated with increasing concentrations of Ang-(1–7), from 10^–8^ to 10^–4^ mol/l (Sigma-Aldrich), alone or in addition to 10^–7^ mol/l Ang II.

The selective MasR antagonist A779 (Abcam, Cambridge, UK) and the selective AT1R antagonist irbesartan (Sigma-Aldrich) were added to fresh media 30 min before treatment with Ang-(1–7).

To investigate the potential cytotoxicity of DIZE, cells were stimulated for 12 h with 10^−5^ and 10^−4^ mol/l DIZE, and counted after trypsin treatment using the trypan blue assay.

### Gene expression analysis

Total RNA was extracted from the human adrenocortical tissues and cells of interest using RNeasy Mini Kit (Qiagen, Milan, Italy) and following a standardized protocol. The quality of the RNA was checked in a Bioanalyzer Agilent 2100 (Agilent Technologies, Santa Clara, California, USA) using the RNA6000 Nano assay (Agilent Technologies). One microgram total RNA was reverse-transcribed with Iscript (Bio-Rad, Milan, Italy) in a final volume of 20 μl.

We obtained absolute quantification of both ACE-2 and ACE-1 mRNA using Digital Droplet PCR system (ddPCR) (Bio-Rad QX200TM; Bio-Rad Laboratories, Segrate, Italy). Droplets were generated using the QX200 Automated Droplet Generator (Bio-Rad Laboratories) and then transferred to a 96-well PCR plate for amplification in the Bio-Rad C1000 (Bio-Rad Laboratories). After PCR, droplets from each sample were streamed in single file through the QX100 Droplet Reader (Bio-Rad Laboratories). Digital Droplet PCR data were analysed with QuantaSoft analysis software (Bio-Rad Laboratories). Absolute levels of the target gene mRNA were expressed as number of copies/μg of RNA.

The relative expression levels of aldosterone synthase (*CYP11B2* gene) and 11β-hydroxylase (*CYP11B1* gene) mRNA were measured with a real-time RT-PCR and calculated by the comparative Ct (2^-ΔΔCt^) method using porphobilinogen deaminase (*PBGD*) as housekeeping gene. Data are presented as mean percentage increase ± SD. Differences observed with real-time RT-PCR were confirmed with digital droplet PCR in a subset of these samples.

Primers for amplification of the genes of interest were designed using ProbeFinder Software (Universal ProbeLibrary, Roche, Monza, Italy, Supplemental Table 2). Each experiment was performed in duplicate and repeated at least five times.

### Immunoblotting

For these studies, samples were obtained from APA tissues (*n* = 3) and the adjacent normal adrenal gland (*n* = 3). Immunoblotting for ACE-2 was performed following a standard protocol. Briefly, tissues were homogenized in a lysis buffer (Thermo Scientific, Milan, Italy) and protein concentration was determined in the soluble supernatant with BCA (Thermo Scientific).

Lysate fraction (50 μg) was separated in a polyacrylamide gel and electro-blotted onto nitrocellulose membrane (Amersham-Hybond EC, GE Healthcare Life Sciences, Milan, Italy). The membranes were blocked for 30 min at room temperature in 5% nonfat dry blocking milk and then incubated overnight at 4 °C with an anti-ACE-2 antibody (diluted 1/500) (NBP1-76614; Novus Biologicals, Milan, Italy).

After washing, membranes were incubated for 1 h with an antirabbit secondary antibody (P0448; Agilent-DAKO, Milan, Italy), after which the band intensity was measured in a VersaDoc Imaging System (Bio-Rad, Milan, Italy). Images were analysed with Image Processing and Analysis in Java (Image J- NIH). ACE-2 expression was normalized to glyceraldehyde 3-phosphate dehydrogenase (GAPDH, #2118; Cell Signaling Technology, Milan, Italy) to adjust for small differences in the amount of loaded protein.

### Formation of angiotensin-(1–7) in human adrenocortical tissues

To prove the feasibility of measuring Ang-(1–7) formation in APA and APA-adjacent tissues, we measured Ang-(1–7) formation in a metabolic assay. Briefly, tissue protein extracts were spiked with Ang II, in the presence and absence of MLN-4760 (Merck-Millipore, Vienna, Austria), an established ACE-2 inhibitor. Positive control samples included recombinant human (rh)ACE-2 (1 ng rhACE-2/μg protein) and a sample of proteins from tissues spiked with rhACE-2; both of which were analysed in the presence and absence of MLN-4760. Protein concentration was measured with the Bradford method and was set equal for each sample.

### Statistical analysis

Statistical analysis was performed with Prism for Mac OS (vers. 9.0, GraphPad Software La Jolla, California, USA). Nonparametric Mann--Whitney test or one-way ANOVA test were used to compare skewed and normally distributed variables, as appropriate.

## RESULTS

### Angiotensin peptides, and *ACE-1* and *ACE-2* gene expressions in adrenal gland

Ang II and Ang-(2–8) were detected by LC-MS/MS in both APA and APA-adjacent tissues (Table 1 and Supplemental Table 3). All the other angiotensin peptides, including Ang I, Ang-(1–7), Ang-(3–8) and Ang-(1–5), were not detectable, because they were below the LLOD of the assay. Ang II and Ang I levels could be measured also in blood from inferior vena cava and in adrenal vein blood draining from APA and the contralateral side, but no differences between sides, even when data were corrected for the selectivity index to avoid a sampling bias because of different proximities of the catheter's tip to the adrenal cortex, were observed.

**FIGURE 1:**
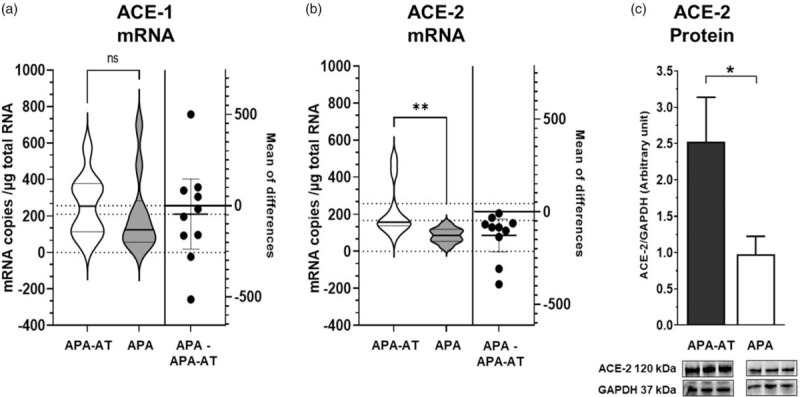
The violin plots show the expression of the angiotensin-converting enzyme 1 (panel a) and the angiotensin-converting enzyme 2 (panel b) in aldosterone-producing adenoma adjacent tissue and in aldosterone-producing adenoma pointing out the frequency distribution of the data. The estimation plot on the right shows the mean difference between tissues; as the confidence interval do not cross 0, the difference was statistically significant for ACE-2 but not for ACE-1 gene expression. Specific mRNAs were quantified with digital droplet PCR (*n* = 10; median, IQR). Immunoblots and histograms show ACE-2 protein expression in APA-adjacent tissue and APA normalized to glyceraldehyde 3-phosphate dehydrogenase (GAPDH) (*n* = 3; mean ± SD) (panel c). ^∗^*P* = 0.03, ^∗∗^*P* < 0.01. ACE-1, angiotensin-converting enzyme 1; ACE-2, angiotensin-converting enzyme 2; APA, aldosterone-producing adenoma; IQR, interquartile range.

Ang-(1–7) and the other angiotensins were undetectable in the blood samples (Table 1). *ACE-2* mRNA levels, measured with ddPCR, and ACE-2 protein were lower in APA than in APA-adjacent tissues, whereas the expression levels of *ACE-1* were similar (Fig. 1).

### Effects of diminazene aceturate on *ACE-2*, *CYP11B1* and *CYP11B2* gene expressions in NCI-H295R

**FIGURE 2:**
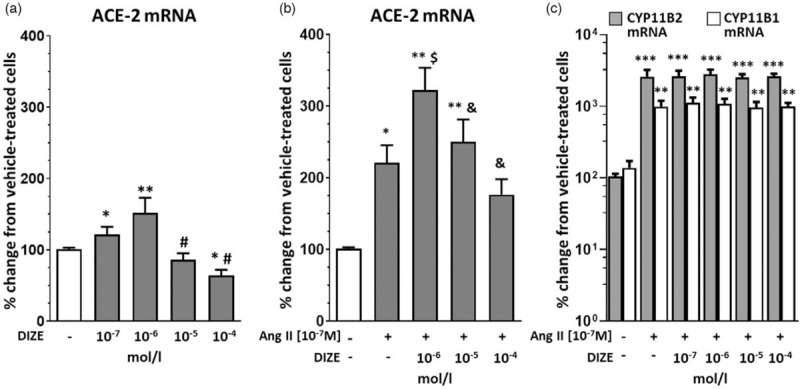
DIZE-induced *ACE-2* gene expression. *ACE-2* gene expression in NCI-H295R cells after exposure to increasing concentrations of DIZE alone, ranging between 10^−7^ and 10^−4^ mol/l (panel a), or in the presence of 10^−7^ mol/l angiotensin II (Ang II) (panel b) (*n* = 5 in duplicate; mean ± SD). DIZE did not blunt Ang II-mediated *CYP11B2* and *CYP11B1* gene expressions. *CYP11B2* and *CYP11B1* gene expressions after treatment of NCI-H295R cells with DIZE at concentrations ranging between 10^−7^ and 10^−4^ mol/l in the presence of 10^−7^ mol/l Ang II (panel c) (*n* = 5 in duplicate; mean ± SD). ^∗∗∗^*P* < 0.0001, ^∗∗^*P* < 0.001, ^∗^*P* < 0.01 compared with vehicle; ^#^*P* < 0.001 compared with DIZE 10^−6^ mol/l; ^$^*P* < 0.01 compared with 10^−7^ mol/l Ang II; ^&^*P* < 0.01 compared with 10^−7^ mol/l Ang II with 10^−6^ mol/l DIZE. DIZE, diminazene aceturate.

We investigated if DIZE, at concentrations ranging from 10^−7^ to 10^−4^ mol/l which were found to be effective *in vitro*[20], affected *ACE-2* gene expression in NCI-H295 cells. We found that at low concentrations (10^−7^ and 10^−6^ mol/l) DIZE increased *ACE-2* gene expression, while at higher concentrations, that is 10^−5^ and 10^−4^ mol/l, it induced a progressive decrease of ACE-2 expression (Fig. 2, panel a).

Therefore, to determine if DIZE blunted Ang II-upregulated aldosterone synthesis, we used it at concentrations ranging from 10^−6^ to 10^−4^ mol/l in the presence of 10^−7^ mol/l Ang II, as a substrate for ACE-2-mediated Ang-(1–7) generation. We found that, although Ang II alone doubled *ACE-2* gene expression (*P* < 0.01) (Fig. 2, panel b), DIZE enhanced this effect at low (10^−6^ mol/l) but not at higher concentrations (Fig. 2, panel b). This was because at 10^−5^ and 10^−4^ mol/l DIZE was cytotoxic, as shown in a cell viability trypan blue assay (Supplemental Table 4).

We also investigated if DIZE affected *CYP11B1* and *CYP11B2* gene expressions at concentrations ranging from 10^−7^ to 10^−6^ mol/l alone or in the presence of 10^−7^ mol/l Ang II. The significant stimulatory effect of Ang II alone on *CYP11B1* and *CYP11B2* mRNA (Fig. 2, panel c) was not blunted by concomitant exposure to nontoxic concentrations of DIZE (Fig. 2, panel c).

**FIGURE 3:**
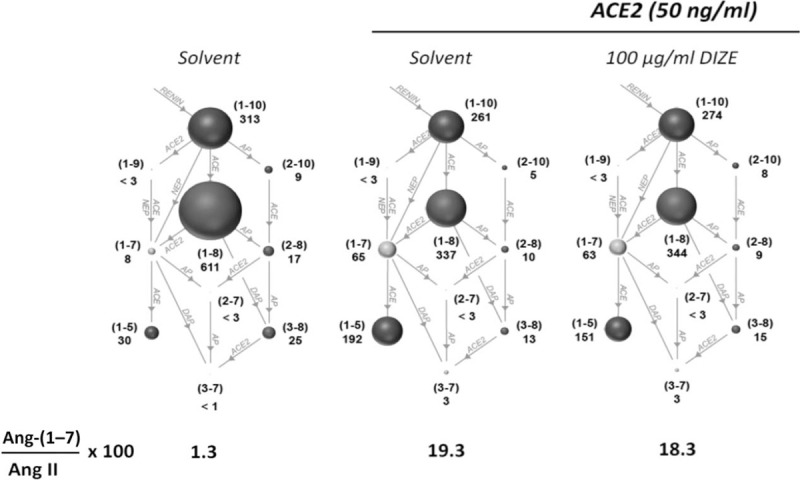
Equilibrium assay quantification of angiotensin peptides in plasma in the absence and presence of recombinant human angiotensin-converting enzyme 2 (50 ng/ml) alone or on top of diminazene aceturate. The similar Ang-(1–7)-to-Ang II ratios in the absence and the presence of DIZE demonstrate that this molecule does not increase the formation of Ang (1–7) by ACE-2. ACE-2, angiotensin-converting enzyme 2; Ang-(1--7), angiotensin-(1–7); DIZE, diminazene aceturate.

We also treated a human heparin plasma pool with rhACE-2 to determine if DIZE could act as an allosteric activator of ACE-2. In a RAS equilibrium analysis, we observed that DIZE did not increase rhACE-2-induced Ang II conversion into Ang-(1–7) (Fig. 3), indicating that DIZE does not act as an allosteric ACE-2 activator under these conditions.

### Effects of angiotensin-(1–7) on *CYP11B2* and *CYP11B1* gene expressions

We investigated if Ang-(1–7) affected *CYP11B1* and *CYP11B2* mRNA as human adrenocortical tissues express not only the AT1-R but also, albeit at much lower levels, the AT2-R and the MasR [9]. We found that, at concentrations ranging from 10^−8^ to 10^−7^ mol/l, Ang-(1–7) did not affect *CYP11B1* or *CYP11B2* mRNA in NCI-H295R cells, as already reported [21].

**FIGURE 4:**
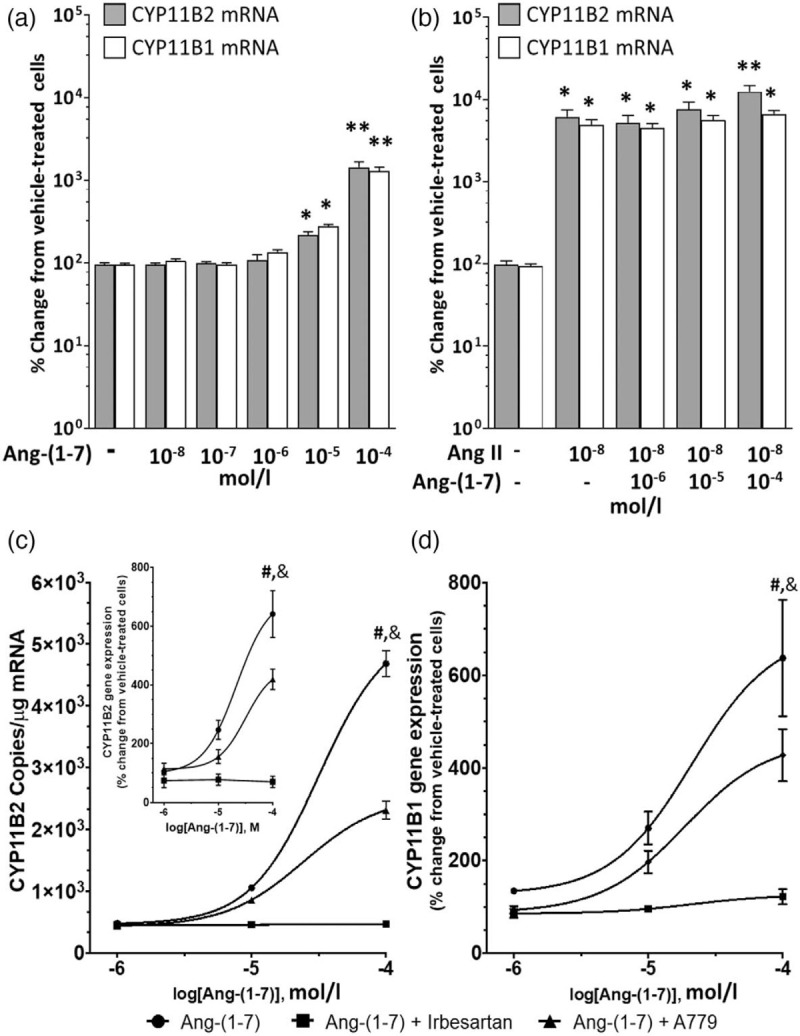
*CYP11B2* and *CYP11B1* gene expressions after treatment of NCI-H295R cells with different concentrations of Ang-(1–7) ranging from 10^−8^ to 10^−4^ mol/l (*n* = 5 in duplicate; mean ± SD). Please note that at high concentration, Ang (1–7) increased *CYP11B2* and *CYP11B1* gene transcripts (panel a). Ang (1–7) did not blunt Ang II-induced expression of *CYP11B2* and *CYP11B1* mRNA. *CYP11B2* and *CYP11B1* gene expressions after treatment of NCI-H295R cells with different concentrations of Ang (1–7) ranging from 10^−6^ to 10^−4^ mol/l in addition to 10^−7^ mol/l Ang II (*n* = 5 in duplicate; mean ± SD) (panel b). Irbesartan abolished Ang (1–7)-mediated *CYP11B2* and *CYP11B1* increase. Dose--response curve of *CYP11B2* (panel c) and *CYP11B1* (panel d) gene expressions after stimulation of NCI-H295R cells with Ang-(1–7) at different concentrations on top of 10^−5^ mol/l irbesartan or 10^−5^ mol/l A779. *CYP11B2* gene expression was analysed with ddPCR and real-time RT-PCR (inset) (*n* = 5 in duplicate; mean ± SD). ^∗∗^*P* < 0.0001 and ^∗^*P* < 0.001 compared with vehicle; ^#^*P* < 0.001 compared with 10^−7^ mol/l Ang-(1–7) with A779; ^&^*P* < 0.0001 compared with 10^−7^ mol/l Ang-(1–7) with irbesartan. Ang-(1--7), angiotensin-(1–7).

At higher concentrations (up to 10^−5^ mol/l) Ang-(1–7) increased *CYP11B1* and *CYP11B2* gene expressions, with a peak effect at 10^−4^ mol/l (13-fold and 14.5-fold for *CYP11B1* and *CYP11B2* mRNA, respectively) (Fig. 4, panel a).

To investigate if Ang-(1–7) could blunt upregulated *CYP11B1* and *CYP11B2*, we first stimulated cells with 10^−7^ mol/l Ang II and then tested the effect of Ang-(1–7) at concentrations ranging from 10^−6^ to 10^−4^ mol/l. These experiments showed that Ang-(1- 7) did not counteract Ang II-upregulated *CYP11B1* and *CYP11B2* mRNA levels (Fig. 4, panel b).

As there are evidences of a weak agonist effect of Ang-(1–7) on AT1R-dependent β-arrestin 2 activation [22,23], this experiment was repeated in the presence of 10^−5^ mol/l irbesartan or of 10^−5^ mol/l A779, the MasR antagonist. A779 blunted the stimulatory effect of Ang-(1–7) high concentrations, whereas irbesartan abolished it, indicating that at high concentration (10^−4^ mol/l) Ang-(1–7) stimulates *CYP11B2* and *CYP11B1* via activation of the AT1R and partially also of the MasR (Fig. 4, panels c and d).

### Angiotensin-(1–7) formation in human adrenocortical tissues

We used LC-MS/MS to quantify the in-vitro generation of Ang-(1–7). Protein extracts derived from an APA and APA-adjacent tissue, which were spiked with rhACE-2 (1 ng rhACE-2/μg protein), showed markedly increased Ang-(1–7) formation (10185 pg/μg protein/h). Treatment with MLN-4760, an ACE-2 inhibitor, reduced this increase by 85% (1519 pg/μg protein/h), indicating that this in-vitro assay was a suitable tool for detecting active ACE-2 in adrenal protein extracts. In unspiked protein extracts derived from APA and APA-adjacent tissue, the amount of Ang-(1-7) generated *in vitro* was 122 ± 22 and 117 ± 40 pg/μg protein/h, respectively (*n* = 10). Endogenous Ang 1–7 formation rates in APA and APA-adjacent tissue remained unaffected by MLN-4760 (APA + MLN-4760: 116.8 ± 33 pg/μg protein/h; APA-adjacent tissue + MLN-4760: 131.3 ± 50 pg/μg protein/h).

## DISCUSSION

ACE-2 has become the target of intense investigative efforts after its identification as the receptor for the SARS-Cov-2 [24,25], the coronavirus that infected over 115 million people and killed over 2.55 million worldwide. The present detection of ACE-2 mRNA in human adrenocortical tissues identifies for the first time the adrenal cortex as a potential target of COVID-19 disease, accounting for occurrence of acute adrenocortical failure contributing to death in a subset of infected patients [26].

This study was set out before the COVID-19 pandemic to seek for a protective role of the noncanonical branch of the renin–angiotensin system in human primary aldosteronism as ACE-2 has been known for years as a key component of the protective branch of the renin–angiotensin system. Specifically, we investigated if ACE-2, its product Ang-(1–7), and activation of the MasR could modulate expression of the cortisol and aldosterone biosynthetic enzymes (*CYP11B1* and *CYP11B2*), thus affecting the main adrenocortical hormones driving pathological changes in multiple diseases.

We used human adrenocortical tissues, which were obtained *ex vivo* with utmost care to avoid peptide degradation, to investigate the tissue amounts of Ang II. In keeping with results of functional studies [27–30], and with the detection of renin [8] and the AT-1R in such tumours [9], we detected Ang II also in some APA and APA-adjacent tissues suggesting that the peptide can play a role in human PA. Interestingly, we did find detectable amounts of both Ang I and Ang II in blood draining from the human adrenal glands of primary aldosteronism patients (Table 1), at similar levels from the adrenal gland harbouring the APA and causing primary aldosteronism, and from the ‘nonculprit’ adrenal gland. These findings can challenge the view that APAs, or at least a subset of them, are autonomous from Ang II.

ACE-2 can generate Ang-(1–9) and Ang-(1–7) from Ang I and from Ang II, respectively, with Ang II being its preferred substrate [31]. Therefore, the findings of ACE-2 mRNA in APA and APA-adjacent tissue suggested a possible modulatory role of the protective arm of the RAS in human primary aldosteronism. However, at variance with this contention we found low levels of Ang I and Ang II in some adrenocortical tissues and no detectable levels in others (Table 1), which might hinder local Ang-(1–7) production, at least to the levels required to play a protective role in PA. Hence, unless ACE-2 levels were markedly high, enough Ang-(1–7) to counteract the enhanced aldosterone secretion that cause high blood pressure and vascular damage in primary aldosteronism could not be generated locally. Our results indicated that these conditions were not met: even though ACE-2 mRNA and protein were detectable in APA and in APA-adjacent tissue, with lower amounts in the former than in the latter tissue (Fig. 1), the absolute amounts of ACE-2 protein were likely too low to ensure Ang-(1–7) formation.

In a functional metabolic in-vitro assay, we could measure MLN-4760-sensitive Ang-(1–7) generation, an unambiguous index of ACE-2 activity when exogenous rhACE-2 was added to protein extracts from our adrenocortical tissues. Importantly, by this assay, in the absence of human recombinant ACE-2, we detected only a scant ACE-2-independent production of the peptide in protein extracts from APA-adjacent and APA tissues.

Noteworthy, the downregulation of ACE-2 in APA paralleled that of the AT2R [9], and collectively suggested no protective effects of the Ang-(1–7)/AT2R axis in hyperaldosteronism. This contention is further supported by our finding that Ang-(1–7) had no effect on *CYP11B1* and *CYP11B2* expressions, even at a concentration (10^−8^ mol/l) (Fig. 4, panel a) much higher than that (10^−12^ mol/l) found in plasma of hypertensive patients [32].

At high concentrations (10^−5^ mol/l and up), Ang-(1–7) stimulated *CYP11B2* via the AT1R agonist, as this effect was abrogated by irbesartan (Fig. 4, panel c). This indicates loss of Ang-(1–7) selectivity for MasR and AT2R at high concentrations in human adrenocortical cells, in line with what seen in rabbit aortae and in rats *in vivo*[33].

The observed lack of modulatory effect of low concentrations of Ang-(1–7) and the stimulatory action at high concentrations in our human adrenocortical cells seem to differ from what found in isolated rat zona glomerulosa cells, where Ang-(1–7) was suggested to blunt Ang II-induced and adrenocorticotropic hormone (ACTH)-induced aldosterone secretion [34]. However, in rodents, the plasma levels of Ang-(1–7) are much higher (in the range 10^−8^ mol/l) [14,15] than in humans where they are in the 10^−12^ mol/l range [32,35,36] which is a quite relevant difference as the affinity of Ang-(1–7) for AT1R (IC_50_ = 10^−5^ mol/l), AT2R [IC_50_ = (2.46 × ^−^10^−7^ mol/l)] and MasR [IC_50_ = (4.75 × 10^−8^ mol/l)] is low [22,37]. Furthermore, in human adrenocortical zona glomerulosa, the expression levels of AT2R and MasR is at least 100-fold lower than the AT1R [9]. These observations could explain the lack of any modulatory effect of Ang-(1–7) on *CYP11B1* and *CYP11B2* Ang II-induced gene expression in our human adrenocortical cells.

We used DIZE, an ACE-2 activator, as a tool to investigate the potential protective effect of enhanced formation of endogenous Ang-(1–7). We discovered that at low concentrations, DIZE markedly raised *ACE-2* mRNA (Fig. 2, panel a), but not ACE-2 activity: when added to recombinant human ACE2 in human heparin plasma, DIZE had no effect on Ang-(1–7) formation and on the Ang-(1–7)-to-Ang II ratio (Fig. 3). On the whole, these findings challenged the view that DIZE is an allosteric activator of ACE-2, at least in human adrenocortical tissues in agreement with previous findings [38]. Of note, like Ang-(1–7), DIZE did not blunt *CYP11B2* and *CYP11B1* expressions (Fig. 2, panel c).

Collectively, our results indicate that the low tissue levels of ACE-2 are insufficient to provide enough Ang-(1–7) generation to modulate aldosterone and cortisol secretion. Thus, they do not support the contention that the protective branch of the renin--angiotensin system can play a major role in the human adrenal cortex. These conclusions are in line with those of Shefer *et al.* but differ from the weak blunting effect of 10^−8^ mol/l Ang-(1–7) on aldosterone secretion and *CYP11B2* gene expression induced by 10^−8^ mol/l Ang II in HAC15 cells [39], a subclone of NCI-H295R cells for reasons that remain unclear. However, in the study by Itcho *et al.*[39], the authors did not use purified or and/or synthetic Ang-(1–7) but conditioned medium obtained after 6 h incubation of HAC15 with Ang II, based on the assumption that cells would have generated Ang-(1–7). Certainly, dissecting the modulatory role of Ang-(1–7) in the human adrenocortical gland requires a careful setup of the experimental conditions, and state-of-the-art techniques, as exploited in our study.

On the whole, while not supporting a protective role of the ACE-2/Ang-(1–7)/MasR/AT2R pathway in the human adrenal cortex and in human primary aldosteronism, our findings can be quite relevant clinically, at a stage when the potential usefulness of strategies aimed at enhancing this protective arm of the renin--angiotensin--aldosterone system are being investigated in multiple cardiovascular diseases.

## ACKNOWLEDGEMENTS

Funding information: this study was supported by research grants from the EU COST-ADMIRE BM1301, ENSAT-HT 633983, FORICA (The Foundation for advanced Research In Hypertension and Cardiovascular diseases, www.foricaonlus.com), the Società Italiana dell’Ipertensione Arteriosa and The University of Padua to Prof. G.P. Rossi, and from Italian Ministry of Health DGRV1113 to professor T.M. Seccia.

### Conflicts of interest

There are no conflicts of intereat

## Supplementary Material

Supplemental Digital Content
