# Supplementary material for: Aldosterone and cortisol synthesis regulation by angiotensin-(1-7) and angiotensin-converting enzyme 2 in the human adrenal cortex
Source: J Hypertens. 2021 Mar 1;39(8):1577–85. doi: 10.1097/HJH.0000000000002816 (PMC9904433; doi:10.1097/HJH.0000000000002816)
Supplement: Supplemental Digital Content [file jhype-39-1577-s001.docx]

**SUPPLEMENTAL MATERIAL**

**Aldosterone and Cortisol Synthesis Regulation**

**by Angiotensin-(1-7) and ACE-2 in The Human Adrenal Cortex**

Brasilina CAROCCIA^a^* PhD, Paul-Emmanuel VANDERRIELE^a^* PhD,

Teresa Maria SECCIA^a^ MD, PhD, Maria PIAZZA^a^ PhD, Livia LENZINI^a^ PhD,

Selene PRISCO^a^ PhD, Francesca TORRESAN^b^ PhD, Oliver DOMENING^c^ PhD,

Maurizio IACOBONE^b^ MD, PhD, Marko POGLITSCH^c^ PhD

and Gian Paolo ROSSI^a^ MD, FAHA, FACC

* Brasilina Caroccia and Paul-Emmanuel Vanderriele contributed equally to this work.

^a^ Specialized Center for Blood Pressure Disorders-Regione Veneto and Hypertension Unit, Department of Medicine-DIMED, University of Padua, Padua, Italy

^b^ Endocrine Surgery Unit, Department of Surgery, Oncology and Gastroenterology, University of Padua, Padua, Italy

^c^ Attoquant Diagnostics, Vienna, Austria

**Running title: Angiotensins in human adrenal gland**

**Corresponding author:**

Professor Gian Paolo Rossi, MD, FACC, FAHA.

DIMED –Clinica dell’Ipertensione Arteriosa

University Hospital

Via Giustiniani, 2

35126 Padova, Italy

Phone: +39-049-821-2279 or 7821

Fax: +39-049-8217873

E-mail: [**gianpaolo.rossi@unipd.it**](mailto:gianpaolo.rossi@unipd.it)

**Supplemental Table 1. Clinical and biochemical profiles of APA patients before and after adrenalectomy.**

|  | **Pre-adrenalectomy** | **Post-adrenalectomy** | **p** |
| --- | --- | --- | --- |
| Patients (n) | 10 | 10 | - |
| Gender (M/F,%) | 60/40 | 60/40 | - |
| Age (yrs) | 52.3±5.9 | 54.2±4.4 | - |
| Systolic BP (mmHg) | 154±13 | 123±8 | <0.001 |
| Diastolic BP (mmHg) | 95±8 | 81±3 | <0.001 |
| Serum K^+^ (mmol/L) | 3.5±0.28 | 4.3±0.54 | <0.001 |
| PAC (ng/dL) | 34.0 (18.5-48.7) | 9.0 (4.6-12.8) | <0.0001 |
| DRC (mIU/L) | 2 (2.0-3.7) | 12.3 (2-19.6) | <0.0001 |
| ARR (ng/mIU) | 78.1 (46-153.4) | 11.6 (3.8-16.9) | <0.0001 |
| AVS Selectivity Index APA side | 31.4 (6.4-46.6) | NP |  |
| AVS Lateralization Index APA side | 19.0 (8.0-55.0) | NP |  |

APA: aldosterone-producing adenoma; ARR: aldosterone–renin ratio; AVS: adrenal vein sampling; BP: blood pressure; DRC: direct renin concentration; PAC: plasma aldosterone concentration; serum K^+^: serum potassium levels. NP= not performed.

Data are presented as mean ± SD, or median [interquartile range] for variables not distributed normally.

**Supplemental Table 2: Sequences of the primer used for gene amplification in real time RT-PCR and ddPCR assays.**

| **Gene** | **Sequences** |
| --- | --- |
| ACE-1  NM_000789.3 | For: 5’- aggagcagaaccagcagaac- 3’  Rev: 5’- tcagcctcatcagtcaccag- 3’ |
| ACE-2  NM_021804.2 | For: 5’- aaagtggtgggagatgaagc- 3’  Rev: 5’- gagatgcggggtcacagtat- 3’ |
| CYP11B2  NM_000498.3 | For: 5’-gtgaccgcaggttgcttt-3’  Rev: 5’-cccttattcctttcccatgc-3’ |
| CYP11B1  NM_000497.3 | For: 5’- ttcagccgccctcaaca- 3’  Rev: 5’- ggatgttcactgatgctgg- 3’ |
| PBGD  NM_000190.3 | For: 5’-tgccctggagaagaatgaag-3’  Rev: 5’- agatggctccgatggtga -3 |

**Supplemental Table 3: Adrenal tissues weights and angiotensin peptides tissue concentration measured in aldosterone-producing adenoma- (APA) adjacent tissue (APA-AT) and in APA.**

| **Patient (Pt)** | **Weight (mg)** | **Ang II [fmol/g]** | **Ang 1-7 [fmol/g]** | **Ang I [fmol/g]** | **Ang III [fmol/g]** | **Ang 1-5 [fmol/g]** | **Ang IV [fmol/g]** |
| --- | --- | --- | --- | --- | --- | --- | --- |
|  |  |  |  |  |  |  |  |
| Pt 1 APA-AT | 68 | 355,9 | <60 | <40 | 54,6 | <25 | <25 |
| Pt 1 APA | 12 | <25 | <60 | <40 | <50 | <25 | <25 |
| Pt 2 APA-AT | 29 | 731,0 | <60 | <40 | 123,3 | <25 | <25 |
| Pt 2 APA | 8 | <25 | <60 | <40 | <50 | <25 | <25 |
| Pt 3 APA-AT | 9 | <25 | <60 | <40 | <50 | <25 | <25 |
| Pt 3 APA | 11 | <25 | <60 | <40 | <50 | <25 | <25 |
| Pt 4 APA-AT | 27 | 167,4 | <60 | <40 | 59,6 | <25 | <25 |
| Pt 4 APA | 14 | 65,3 | <60 | <40 | <50 | <25 | <25 |
| Pt 5 APA-AT | 69 | <25 | <60 | <40 | <50 | <25 | <25 |
| Pt 5 APA | 24 | <25 | <60 | <40 | <50 | <25 | <25 |
| Pt 6 APA-AT | 22 | 1701,7 | <60 | <40 | 1078,4 | <25 | <25 |
| Pt 6 APA | 22 | 77,0 | <60 | <40 | 124,3 | <25 | <25 |
| Pt 7 APA-AT | 26 | 150,6 | <60 | <40 | <50 | <25 | <25 |
| Pt 7 APA | 19 | 111,9 | <60 | <40 | <50 | <25 | <25 |
| Pt 8 APA-AT | 18 | <25 | <60 | <40 | <50 | <25 | <25 |
| Pt 8 APA | 25 | <25 | <60 | <40 | <50 | <25 | <25 |
| Pt 9 APA-AT | 16 | 122,5 | <60 | <40 | <50 | <25 | <25 |
| Pt 9 APA | 18 | <25 | <60 | <40 | <50 | <25 | <25 |
| Pt 10 APA-AT | 26 | 43,1 | <60 | <40 | <50 | <25 | <25 |
| Pt 10 APA | 25 | 195,2 | <60 | <40 | <50 | <25 | <25 |

**Supplemental Table 4: DIZE effects on cells viability**

| **Treatment** | **Cell viability (in %)** |
| --- | --- |
| Vehicle | 100 |
| DIZE 10^-7^ M | 98 ± 2 |
| DIZE 10^-6^ M | 99 ± 1 |
| DIZE 10^-5^ M | 85 ± 1 |
| DIZE 10^-4^ M | 83 ± 5 |
